# Gestational hormone trajectories and early pregnancy failure: a reassessment

**DOI:** 10.1186/s12958-018-0415-1

**Published:** 2018-10-11

**Authors:** Paul G Whittaker, Courtney A Schreiber, Mary D Sammel

**Affiliations:** 1 Department of Obstetrics and Gynecology, Penn Family Planning and Pregnancy Loss Center, Perelman School of Medicine, University of Pennsylvania, 1000 Courtyard, 3400 Spruce St, Philadelphia, PA 19104 USA; 2 Department of Biostatistics and Epidemiology, Center for Clinical Epidemiology and Biostatistics, Perelman School of Medicine, University of Pennsylvania, Philadelphia, PA 19104 USA

**Keywords:** Oestradiol, hCG, Progesterone, Miscarriage

## Abstract

**Background:**

Studies have commonly assessed the endocrinolgical status of women once miscarriage is threatened or suspected; few studies have explored the antecedent hormonal environment or used a longitudinal strategy. Using refined statistical techniques, we sought to re-evaluate whether gestational hormone trajectories in early pregnancy can identify future miscarriage in asymptomatic pregnancies.

**Methods:**

This prospective cohort study followed 105 women over-conception; 72 had normal term pregnancy outcomes while 33 experienced early pregnancy failure between 35 and 115 days of gestation. Participants attended a pre-conception and antenatal clinic at Newcastle University, United Kingdom (UK). Evaluation methods included ultrasound, clinical assessments of pregnancy progress and serial measurements of gestational hormones by radioimmunoassays. Linear mixed-effects regression analysis examined hormone relationships with pregnancy outcomes.

**Results:**

Detailed longitudinal illustration of gestational hormones, antecedent to miscarriage indications, revealed early pathophysiological trends. In particular, oestradiol showed as marked a deviation from normal as progesterone before miscarriage was evident, reflecting a deficiency in the ovarian response to rising human chorionic gonadotrophin (hCG) levels. Regression analysis provided equations for gestational hormone slopes that significantly differentiated asymptomatic women with subsequent early pregnancy failure, compared to women with normal term pregnancies. Both progesterone and oestradiol displayed negative mean slopes in pregnancies destined for failure; in this group, both human placental lactogen (hPL) and hCG revealed mean positive trajectories that imitated normal pregnancies but at slower rates of increase.

**Conclusions:**

Oestradiol, progesterone and hCG trajectories, from 50 days of gestation, have good potential for revealing pathophysiology and for identifying which asymptomatic pregnancies are destined for subsequent failure. In asymptomatic patients where there is concern about viability and ultrasound diagnosis is ambiguous, a combined hormonal profile could contribute to guiding patient care decisions.

## Background

Early pregnancy failure (EPF) is a common event in human pregnancy, accounting for 10–20% of recognized pregnancies [1]. Progesterone and human chorionic gonadotrophin (hCG) are the most common serum markers for assessing pregnancy viability when ultrasound findings are inconclusive [2]. Progesterone, initially from the corpus luteum, is essential for maintaining early pregnancy [3]. HCG, from villous trophoblast, supports luteal progesterone production, and facilitates the shift of progesterone and oestradiol production to the placenta around 8–9 weeks of pregnancy.

Clinical studies have commonly assessed the endocrinolgical status of women once EPF is threatened or suspected, with the aim of predicting outcomes and the most appropriate clinical management. Recent systematic reviews and meta-analyses have indicated that a single measurement of progesterone, oestradiol or hCG can predict pregnancies likely to continue among women with threatened miscarriage [4]. However, these authors acknowledge that choosing a discriminatory value to predict viability is difficult and gestational age specific nomograms would be required. Serial hCG measurements have been documented to predict viable intrauterine pregnancy in symptomatic patients with a pregnancy of unknown location [5–7].

Few studies have explored the hormonal environment antecedent to symptoms of EPF. We undertook a prospective serial study which measured maternal hormones before, during and after both normal pregnancy and EPF, and have made a reassessment (using up-to-date regression analysis) of our previously published data [8], to further clarify the possible hormonal associations with poor pregnancy outcome.

## Methods

A prospective cohort of women was recruited at the pre-conception clinic at Newcastle University, UK, over a 7 year period from 1978 to 1985. The main criterion for inclusion was a willingness to attend for weekly visits prior to conception, biweekly visits during the first trimester, and monthly visits during the remainder of pregnancy and until 3 months post-delivery. We have selected those 105 pregnancies who had uncomplicated past medical and obstetric histories, and who ovulated spontaneously. In this cohort, 72 women had normal pregnancies without medical complications, and were delivered of single, live, healthy infants after 37 completed weeks’ gestation. The other 33 women experienced early pregnancy failure during the study, between 35 and 115 days of gestation (from their last menstrual period). All blood samples were taken before any EPF symptoms developed. The mean time elapsed between last blood sample and EPF delivery was 12 (SD 8) days. Ultrasound showed that eight women developed an obvious foetus with a detectable heartbeat before EPF, while 25 had a gestational sac but no foetal echoes were ever detected. Clinical details of these 105 women and the hormone assay methods for progesterone, oestradiol, hCG and hPL have been published previously [8]. At each clinic visit, blood samples were obtained from the women in the morning after an overnight fast. Not all women attended on every occasion due to working practices, holidays, et cetera. The average number of samples per woman was 3.6 (range 1–8); all but one woman provided two or more samples. This research had the approval of Newcastle University’s Ethics Committee in 1978 and all the women gave written informed consent.

Log (base10) transformation of the hormone values was used to reduce the skewness of their data distribution. Reference ranges for normal hormone values were constructed as the geometric mean (± 2 SD) of data groups centred on days 21, 28, 42, 56, 70 and 84 days of gestation. The association between hormonal changes over time and pregnancy outcomes was assessed using t-test, chi-square test, and regression where appropriate. The dynamics of hormone levels over time were modelled using linear and quadratic mixed-effects regression which allow for within and between-subject variation to enhance precision in slope estimates of hormonal change [9]. These models use random effects to account for each participant having contributed repeated hormone measurements and allow for variation in the number and timing of observations. Random effects models estimate a population average curve by aggregating estimated hormone profiles from each individual subject [5]. This regression analysis can better distinguish the hormone trajectories of the two groups using all their data (our original paper [8] analysed ‘within patient’ differences across select time-points but couldn’t include all patients at each time point due to missing data).

Our regression models assumed both random intercept and slopes, along with unstructured covariance. The unstructured covariance fit the data best as evaluated using Akaike Information Criteria [10]. The regression analysis also facilitated the evaluation of adverse pregnancy outcome as an influence on hormonal changes. The statistical software packages SPSS 22 (IBM) and Stata 14 (Statacorp LP) were used.

## Results

### Clinical characteristics of study participants

Table 1 shows that participants with EPF had on average a higher non-pregnant BMI (*P* = 0.014) than participants with normal pregnancies; the average difference of 1.6 kg.m^2^ is equivalent to about 4 kg for a woman of average height. In normal participants, we assessed the relationship between the non-pregnant BMI and hormone levels and trajectories between 6 and 12 weeks and found no significant correlations (all *P* > 0.05). Participants with EPF were more likely (*P* = 0.024) to be multigravid than participants with normal pregnancies. In normal participants, we assessed the relationship between gravidity and hormone levels and trajectories between 6 and 12 weeks and found no significant associations (all *P* > 0.05).

**Table 1:** Clinical characteristics of study participants

	Normal Pregnancy	Early Pregnancy Failure
	*N* = 72	*N* = 33
Age (years)	29.5 (3.5)	30.8 (4.7)
BMI (kg/m^2^)	22.3 (2.7)	23.9 (3.3)*
Gravidity		
1	22 (31%)	3 (9%)
≥ 2	50 (69%)	30 (91%)*
Previous EPF (if previously parous)	20 (40%)	17 (57%)
Gestation at delivery (completed weeks since last menstrual period)	40 (37–42)	10 (6–16)
Birthweight (kg)	3.45 (2.60–4.50)	n/a

Results are mean (SD), median (range) or n (%) as applicable. Group comparisons used the Fisher exact test or t-test as appropriate. *P < 0.05

### Individual hormone trajectories

**Fig. 1:**
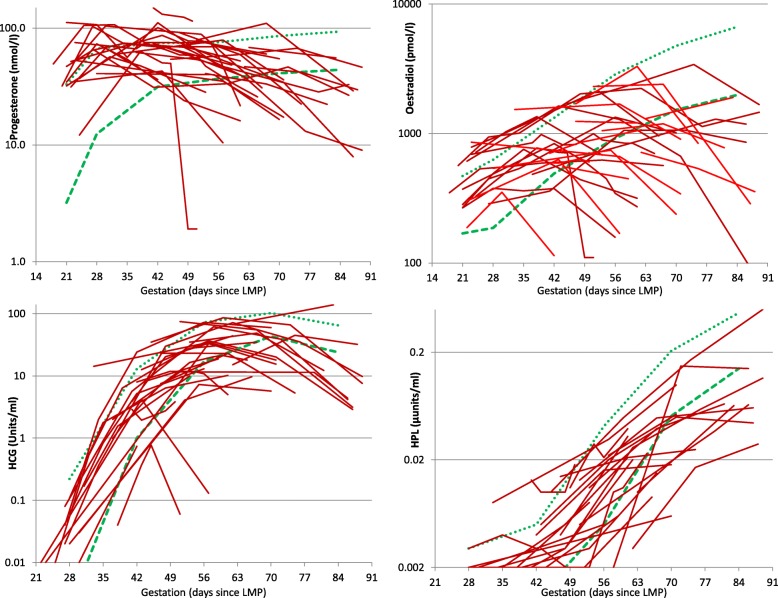
Serum Progesterone, Oestradiol, hCG and hPL in early pregnancy. Dotted green lines indicates the geometric mean value for normal (term) pregnancies, dashed green line indicates the lower 2xSD boundary of normal (term) pregnancy values. Red lines indicate individual values for pregnancies resulting in early failure

Within-woman changes in hormone levels over time are shown in Fig. 1. For EPF women, hormone levels were all within normal limits (within 2 SD of the normal female mean) up to 44, 33, 38 and 49 days of gestation for progesterone, oestradiol, hCG and hPL respectively. Thereafter, though many EPF women showed a variable trend, a majority had normal progesterone, oestradiol, hCG and hPL levels up to 66, 60, 63 and 63 days respectively. A substantial minority of EPF women had hormonal levels beyond 50 days that were below the normal range, even though none were yet symptomatic of EPF.

### Regression analysis

Table 2 shows regression coefficients which describe hormone trends over time of progesterone and oestradiol and tests whether the longitudinal trends differ by Outcome (EPF or normal pregnancy). The significant interaction terms (Outcome*Gestation) indicate that the rates of increase of these two hormones, together with hPL, were significantly different between EPF and normal gestation. Because the hCG profiles have a reversal between 8 and 12 weeks, an enhanced description of the trends in hCG levels was achieved by breaking down the regression analysis into two time-windows. A quadratic function was included in the hCG regression model for the portion of rising hCG levels (days 29 to 74) and a simple linear model used for the descending portion (days 75 to 91). Table 3 shows that for hCG, the Outcome and Outcome*Gestation interaction covariates were significant in both the rising and falling components of hCG trends, which suggested EPF was associated with an overall difference in hCG trends.

**Table 2:** Association between early gestation hormone trajectories and Early Pregnancy Failure

Covariate	Progesterone	Oestradiol	hPL
Gestation (days)	0.002 (0.001)***	0.016 (0.001)***	0.045 (0.001)***
Outcome	0.463 (0.075)***	0.572 (0.105)***	0.159 (0.167)ns
Outcome*Gestation	−0.013 (0.001)***	− 0.020 (0.002)***	− 0.012 (0.002)***

Regression coefficients (SE) were from linear mixed effects models (Stata). ***p < 0.001, ns *p* > 0.05. Data included serial hormone levels (log transformed) from 33 women with early pregnancy failure (96 observations) and 72 women with normal pregnancy outcomes (280 observations). Gestation was limited to between 29 and 91 days from last menstrual period. hPL – human placental lactogen

**Table 3:** Association between hCG trajectories in early gestation and Early Pregnancy Failure

Covariate	Timeframe (days from last menstrual period)	
	29–74 days	75–91 days
Gestation (days)	0.294 (0.015)***	− 0.017 (0.002)***
Gestation Squared	− 0.0023 (0.0001)***	
Outcome	1.82 (0.75)*	1.03 (0.44)*
Outcome*Gestation	−0.073 (0.027)**	−0.021 (0.006)***
Outcome*Gestation Squared	0.0005 (0.0002)*	

Regression coefficients (SE) shown were from linear mixed effects models (Stata). ***p < 0.001, **p < 0.01, *p < 0.05. Data included serial hormone levels (log transformed) from 33 women with early pregnancy failure (96 observations) and 72 women with normal pregnancy outcomes (280 observations). Gestation was limited to between 29 and 91 days from last menstrual period. hCG – human chorionic gonadotrophin

Within the EPF group, hormone trajectories were not associated with the timing of eventual pregnancy loss (day of Delivery). Analysis (data not shown) indicated that, for all four hormones, the regression coefficients for the Delivery and Delivery*Gestation interaction covariates were not significant.

### Graphs of regression trajectories

**Fig. 2:**
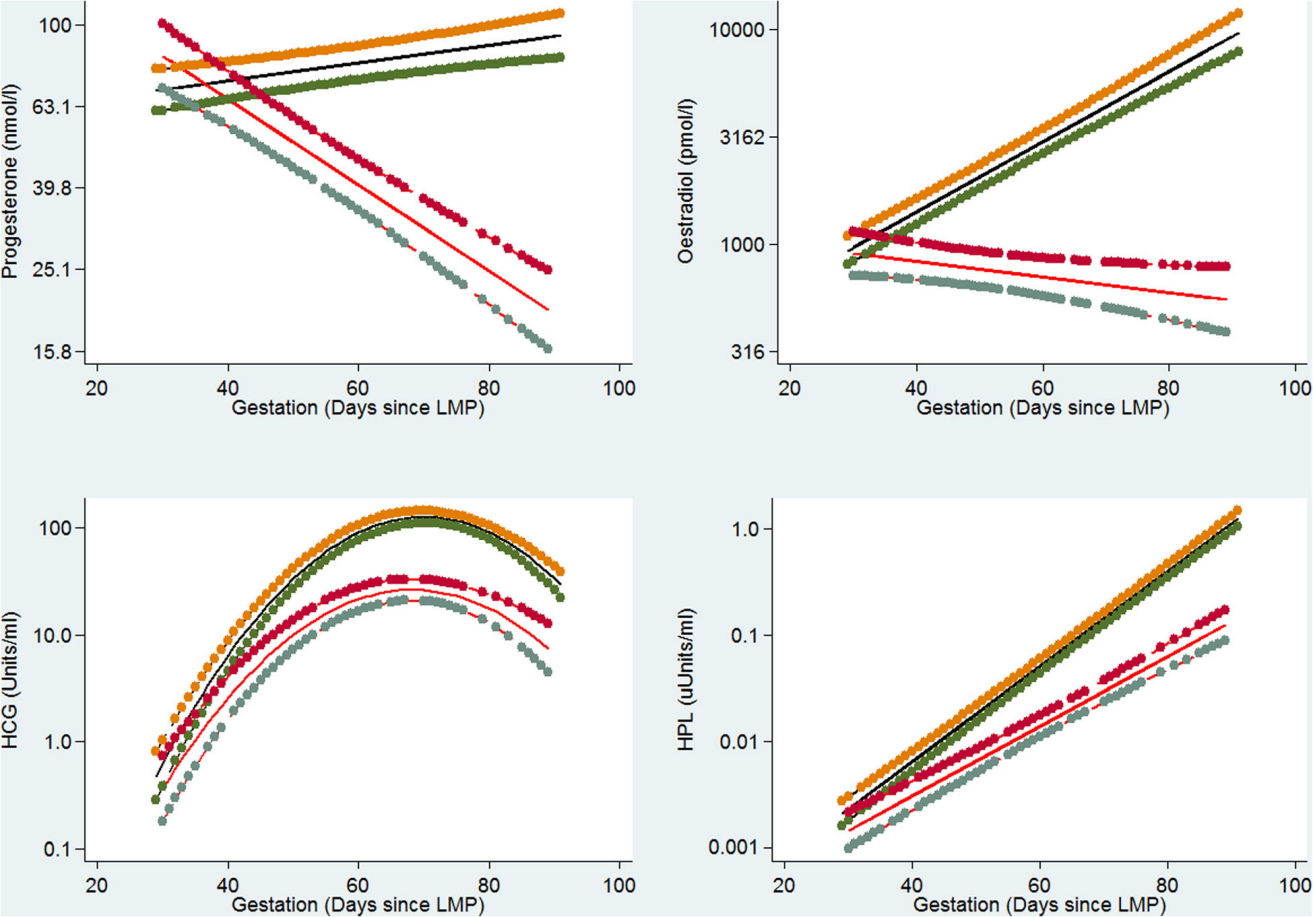
Regression profiles of serum Progesterone, Oestradiol, hCG and hPL in early pregnancy. Mean regression lines are shown as black for normal pregnancies and red for pregnancies ending in miscarriage. The 95%CI of the mean lines are denoted by connected dots

The graphical representation of hormone regression slopes are shown in Fig. 2. These show that for progesterone, hCG and hPL before 45 days (and oestradiol before 40 days), mean hormone levels overlapped between normal and EPF pregnancies. Beyond 50 days of gestation, both mean hormone levels of all four hormones, and their rate of change, appeared markedly different between normal and EPF pregnancies. The graphs indicate that for EPF pregnancies, both progesterone and oestradiol showed negative mean slopes. With pregnancies destined for EPF, both hPL and hCG showed mean positive trajectories that mirror normal pregnancies but at slower rates of increase. Peak mean hCG levels were reached at about 70 days in both groups and thereafter declined.

## Discussion

### Main findings

Our longitudinal analysis clearly shows that, in asymptomatic women, all four hormones we measured displayed initial overlap between normal pregnancies and those destined for EPF. From around day 50 from LMP, we noted a clear divergence in trajectories. Hormone profiles of women with EPF showed that placental production of hCG and HPL was rising in early pregnancy but at a lower rate than normal. Levels of progesterone and oestradiol, from the corpus luteum and normal around day 42 (6 weeks), declined thereafter suggesting a lack of placental steroidogenesis. Of note is that oestradiol showed as marked a deviation from normal as progesterone, indicating a deficiency in the ovarian response to rising hCG levels [3]. We have noted previously the complexity of modelling the hCG levels. Our earlier analysis [8] of the within-woman hCG trends showed that the average upward increments between three to 6 weeks and six to 8 weeks in EPF patients were significantly less than the normal upward rise. However, the average decrement from 8 weeks to 12 weeks in EPF patients was not different from the normal decline over this time span [8]. Our regression analysis now reveals that there is significant variation in declining hCG slopes attributable to outcome.

### Strengths and limitations

Strengths of this study include the large patient number and frequent sampling, its prospective nature, a study population that included only asymptomatic women, and the combination of hormonal markers of both ovarian function (progesterone, oestradiol) and trophoblast function (hCG, hPL) [3]. One limitation of this study is that the day of final pregnancy demise is heterogeneous across patients, which increases variability in the data. Also, the potential impact of aneuploidy on hormone levels is unknown. When assessing current relevance of the findings, it is conceivable that there has been a secular shift in gestational hormone levels, perhaps related to increasing body mass index over the past three decades, though there is no evidence that the distinction between normal and problem pregnancies would be obscured thereby.

### Interpretation

Recent reviews have assessed the value of hormonal assessment of EPF [1, 2, 4, 6, 7]. The utility of the association of a slow rise in hCG and/or a low serum progesterone with EPF is predominantly derived from studies of symptomatic women and has rarely used longitudinal data over the first trimester. There are only a couple of published studies of asymptomatic women who develop EPF that have included oestradiol. Asymptomatic IVF patients were evaluated at weekly intervals until 11 weeks and the report [11] noted that a single progesterone, oestradiol or hCG, at or after 7 weeks, was significantly lower in pregnancies destined for EPF. A study [12] evaluated asymptomatic patients before 11 weeks (mean gestation 7 weeks) and noted that a single progesterone or hCG, but not a single oestradiol, was significantly lower in pregnancies destined for EPF. However, a recent meta-analysis [4] has reinforced the value of oestradiol measurement in predicting the outcome of threatened miscarriage. Although the value of hPL as a marker of ectopic pregnancy, independent of hCG, has been questioned [13], the real question as to its utility remains the very low hPL values seen before 8 weeks gestation in many normal pregnancies.

## Conclusion

Our data suggest that a combination of oestradiol, progesterone and hCG trajectories have good potential for identifying EPF in early pregnancies, from 50 days of gestation onwards. In asymptomatic patients where there is concern about viability and ultrasound diagnosis is ambiguous, a hormonal profile may be a useful addition to ultrasound diagnosis and follow-up in guiding patient care decisions [14, 15]. Future research would evaluate these markers, as well as novel markers [4, 13], in a new group of women with suspected failure of intrauterine gestation, to confirm that these hormonal marker trajectories can discriminate non-viability of an early pregnancy with high specificity and examine whether differences in hormone profiles are associated with the timing of pregnancy loss.

## Abbreviations

EPF: Early pregnancy failure
hCG: Human chorioinic gonadotrophin
hPL: Human placental lactogen
